# P-1701. Characterizing 24-Hour Pharmacist Response to Rapid Multiplex Polymerase Chain Reaction (rmPCR) Blood Culture Results

**DOI:** 10.1093/ofid/ofaf695.1873

**Published:** 2026-01-11

**Authors:** Noah Sanford, Rachel Friend, Mary Kate Lackey, Elizabeth W Covington, Sarah G Gunter

**Affiliations:** East Alabama Medical Center, Auburn, Alabama; East Alabama Medical Center, Auburn, Alabama; Auburn University Harrison College of Pharmacy, Auburn, Alabama; Auburn University Harrison College of Pharmacy, Auburn, Alabama; East Alabama Health, Opelika, AL

## Abstract

**Background:**

Rapid molecular polymerase chain reaction (rmPCR)-based blood cultures with pharmacist-driven response have been studied with differing means of communication and limited hours of pharmacist coverage. Our study aimed to characterize 24-hour pharmacist response to rmPCR blood culture results with a focus on response differences between shifts and time to optimal antibiotics.

**Table 1:**
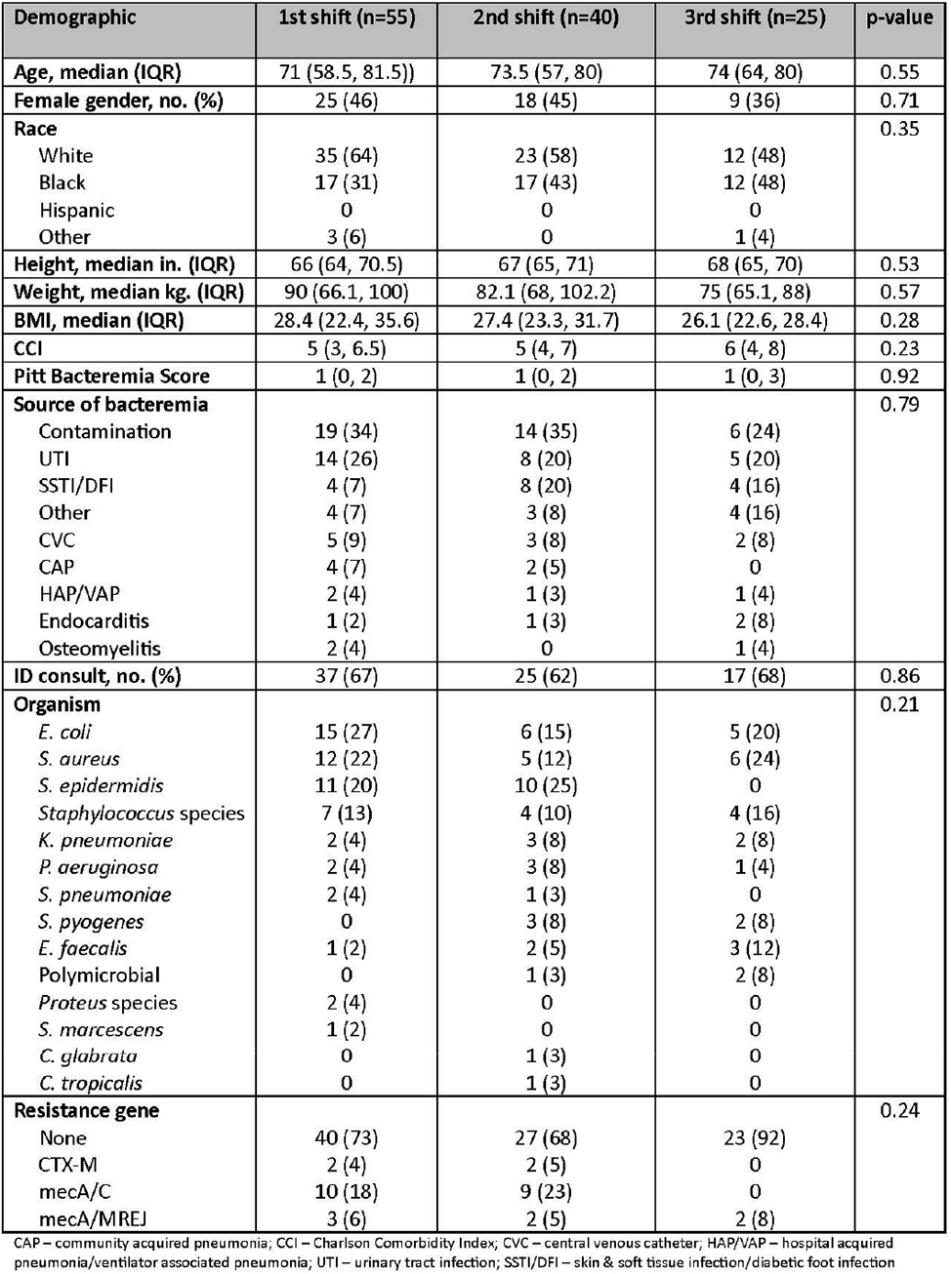
Baseline Characteristics

**Table 2:**
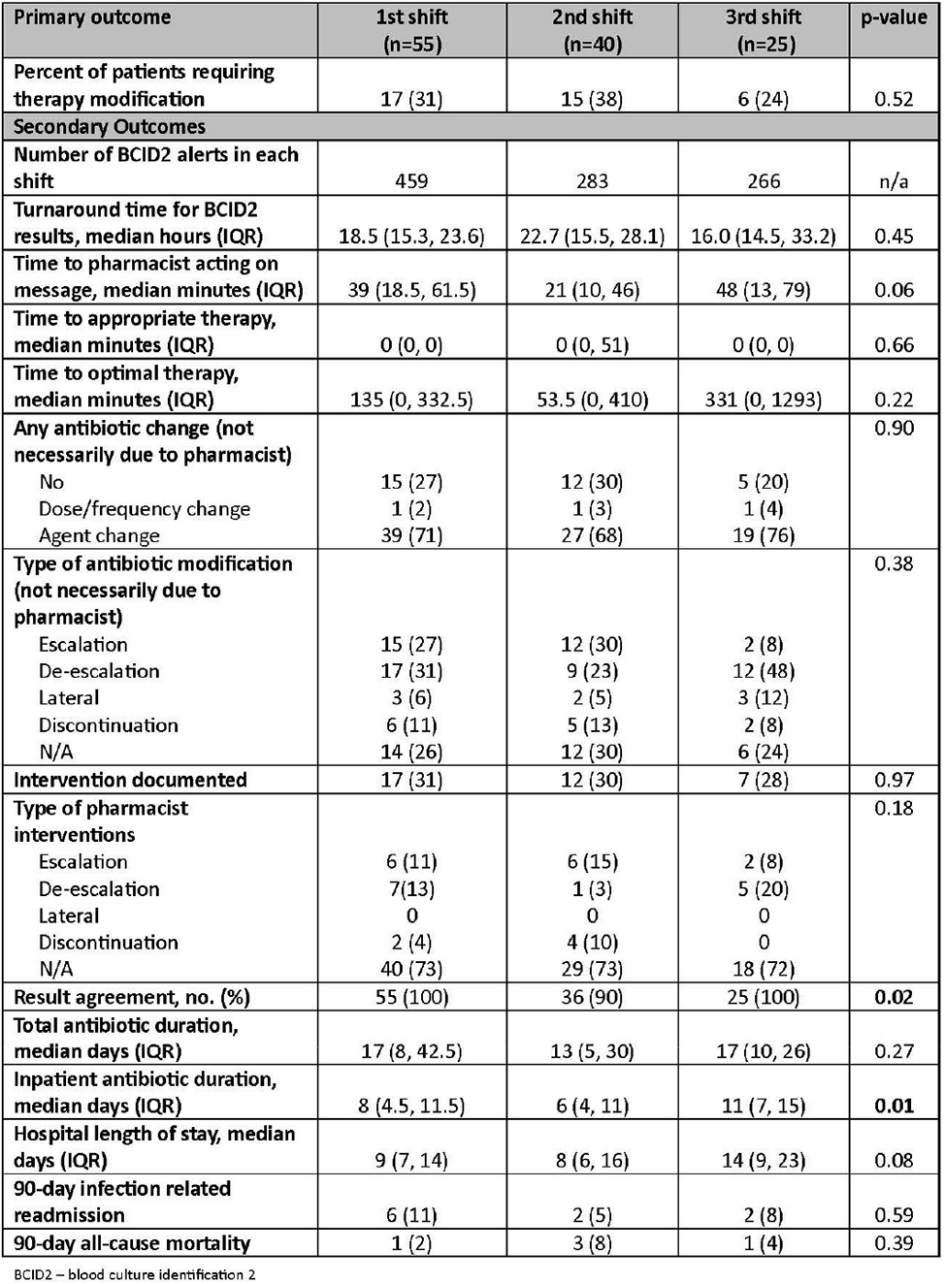
Primary and Secondary Outcomes

**Methods:**

This retrospective chart review included patients ≥19 years old with positive blood cultures admitted to East Alabama Medical Center. Patients were excluded if they were pregnant, incarcerated, discharged or transitioned to comfort care within 8 hours of blood culture notification, if they died within 24 hours of blood culture notification, or had rmPCR results with *Streptococcus* species, *Streptococcus agalactiae*, *Cryptococcus*, or *Enterobacterales*. Pharmacists were alerted to positive rmPCR results via electronic medical record task list and made antibiotic recommendations based on in-house guidelines. Shifts were divided as follows: first (0700-1459), second (1500-2259), and third (2300-0659). The primary outcome was the percentage of patients requiring therapy modification. Secondary outcomes included time to pharmacist intervention and time to optimal therapy.

**Table 3:**
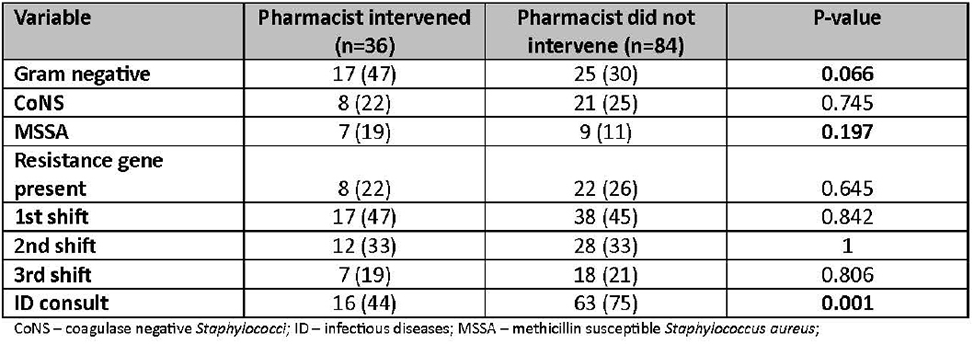
Bivariate Analysis

**Results:**

In total, 120 patients were included: 55 in first shift, 40 in second shift, and 25 in third shift. Baseline characteristics were similar among the three groups. There was no difference in the primary outcome: 17 patients required therapy modification in the first shift group (31%), 15 (38%) in second shift, and 6 (24%) in third shift (p=0.516). There was no difference in time to pharmacist intervention (p=0.062) or time to optimal therapy (p=0.219) across shifts. Pharmacists were more likely to intervene on patients with methicillin-susceptible *Staphylococcus aureus* (odds ratio [OR] 5.7, 95% confidence interval [CI] 1.60 to 20.74) and less likely to intervene on patients with an infectious disease consult (OR 0.21, 95% CI 0.084 to 0.530). Pharmacist shift was not associated with likelihood of intervention.

**Conclusion:**

Our study showed a similar need for therapy modification and similar response times across shifts, highlighting the value of 24-hour pharmacist review of rmPCR results.

**Disclosures:**

All Authors: No reported disclosures

